# Resistance of soil to penetration as a parameter indicator of subsolation in crop areas of sugar cane

**DOI:** 10.1038/s41598-021-91186-3

**Published:** 2021-06-03

**Authors:** Aline Barbosa Arruda, Rodrigo Fernandes de Souza, Gustavo Henrique Mendes Brito, Jadson Belém de Moura, Manoel Henrique Reis de Oliveira, José Mateus dos Santos, Sandro Dutra e Silva

**Affiliations:** 1 Postgraduate Program in Agronomy, Goias Federal University, Goiânia, Brazil; 2 Evangelical Faculty of Goianésia, Goiás, Brazil; 3 Program in Natural Resources of the Cerrado, State University of Goiás, Goiás, Brazil; 4 Evangelical University of Goiás, Goiás, Brazil

**Keywords:** Plant sciences, Biofuels

## Abstract

Sugarcane is a very important economic crop that relies heavily on agricultural machinery, which contributes to soil compaction and a consequent decline in productivity. Subsoiling operation reduces the problems caused by compression; however, it is necessary to know its location and intensity. Accordingly, the aim of this work is to present a compression diagnostic method based on soil resistance to penetration as the parameter that indicates need for intervention in the subsoil. Measurements of penetration resistance was carried out in areas of sugarcane, located in the municipalities of Goianésia, Barro Alto and Santa Isabel, in the Brazilian state of Goiás. The Falker penetrometer (PLG 1020) was used, adjusted to a maximum depth of 40 cm and adopted as a critical resistance value of 4.0 MPa. The data were interpolated using kriging and adjusted in AutoCAD 2013 (Autodesk). The methodology proved effective in areas of compacted soil, and the surface layer had less resistance. The reduction in soil preparation was 96.54% and when considering the topographic adjustments, the reduction was 74.07%, showing the viability and importance of the diagnosis to show the proper management.

## Introduction

Sugarcane (*Saccharum officinarum*) is one of the most important crops for the global economy. It is a valuable source of income, employment and supports economic development^1,2^. In the Brazilian socioeconomic scenario, sugarcane occupies a remarkable position. Brazil is the world's largest producer of this raw material, which is the primary industrial input to produce sugar^3,4^. Moreover, it is also used in the production of ethanol, used as biofuel, and utilized as biomass production for renewable energy^5^. Because of its importance to the national economy, sugarcane production has expanded into almost all Brazilian states and biomes. The data was collected in the Brazilian State of Goiás, located in the Cerrado biome. The Cerrado is the second largest Brazilian biome, extending over an area of 2,045,064 km^2^ ^6,7^. Only the Amazon surpasses the Cerrado in terms of total area. Currently, the Cerrado is considered to be the last agricultural frontier in the Americas^8–12^.

Within this context, Goiás stands out as a state of central importance, due to the expansion of its planted area, as well as its increasing production and productivity^4,13^. In the municipality of Goianésia, for instance, sugarcane cultivation began in the late 1960s. Nowadays, it is one of the region’s most important agro-industrial activities, and the local economy benefits through higher tax revenues and job creation^14^.

The most important sugarcane varieties originate from Oceania (New Guinea) and Asia (India and China), whereas the cultivated varieties in Brazil and the world are multispecific hybrids^15^. Belonging to the Poaceae family and to the class of monocotyledons, sugarcane has a long life-cycle with a span of 5–7 years^2^. Some of its main characteristics are barb type inflorescence, stem growth in culms, leaves with silica blades on their edges and an open hem^15^. In spite of the long life-cycle of sugarcane, an important feature is the sufficient development of its root system^2^ because it provides increased productivity of culms^16^, which are the parts of greater commercial interest.

Root system development may be limited by the physical conditions of soil, especially regarding compression. Soil compression increases resistance to the plant’s root development and consequently decreases agricultural productivity^17^.

Even when the soil contains adequate nutrients, plants cannot properly uptake nutrients if there is compaction of the soil that prevents the water absorption. This is a consequence not only of the reduction of free spaces in soil, but also of the reduction of oxygen in the rhizosphere, which may limit the plants’ metabolic processes. Moreover, compaction can limit the roots ability to absorb water and nutrients^18^.

Through the intensification of agricultural mechanization, problems of compaction in agricultural soils are increasingly problematic. These problems are a consequence of the intensive and extensive traffic of machinery and other heavy implements, especially when conditions favor compression^17,19^.

The negative effects of compression can be minimized by breaking compacted layers through subsoiling operations, which must be performed only where it is really necessary at an adequate depth^2^. Subsoiling is often carried out without reference to studies that could properly guide the actual need of such procedure^2^.

As a method for diagnosing compaction zones and their intensity, some authors recommend the use of penetrometers. These devices can indicate the resistance mounted by the soil against the penetration of a conical tip, which can simulate the resistance that the ground offers to the penetration of roots^2,20–22^.

The evaluation of resistance against penetration through the use of a penetrometer is indicated when soil–water content lies near field capacity, since it is this condition that produces the best correlation between soil bulk density and root growth of plants^23–25^. Soils cultivated with sugarcane cultivation are likely to face problems regarding root development of the crop when resistance against penetration is higher than 4.0 MPa, which is considered a critical figure^2,23,26,27^. Thus, it is necessary to carry out such operations to break up compacted layers. Such operations can reduce damage to the crop and promote growth.

Considering the above, this work aims to present the diagnostic method of soil compaction, based on soil penetration resistance, as a parameter that indicates a real need of subsoiling operations in areas of sugarcane cultivation.

## Material and methods

The experiment was carried out from September 2016 to May 2017 in areas of commercial sugarcane cultivation in the municipalities of Goianésia, Santa Isabel and Barro Alto, in the state of Goiás, located in the Center-West region of Brazil, in the Cerrado biome. This study area’s climate is predominantly tropical, with a notable division between two well-defined seasons, with a hot and humid summer and a dry and cool winter. Average temperature varies between 18 and 26 °C^28^.

The study area consisted of 1038.73 ha dedicated to sugarcane replanting. All these areas have more than 5 years of sugarcane cultivation. The soil types of the evaluated areas were Inceptisols and Oxisols. The methods for this study were carried out in five stages, which are detailed below.

### Elevating soils to field capacity

To conduct the soil at field capacity, portable drips were made in plastic polyethylene containers with a volume of 20 L. Metal framework recovered from stools was also used. Drippers were used at all sampling points.

After following the test methods recommended^29^, the drippers were standardized for a flow of 4 L per hour, for a period of 5 h for the consumption of 20 L of water in the containers. Thus, wet bulbs with a minimum depth of 40 cm were formed in all evaluated soils.

The drip technique was chosen because it keeps soil moisture always close to the field capacity, which occurs due to the high frequency and low volume of humid soil^30^.

### Penetration resistance sampling

Data collection was conducted in humidified points until field capacity, considering a point per hectare^2^. The sampling points were properly georeferenced in UTM coordinates, with the WGS 84, Zone 22 South, via GPS navigation device.

Penetration resistance values were obtained from a penetrometer from the Falker brand (model PLG 1020), with automatic measuring system conditioned to a nominal speed of 3.0 m s^−1^, and with a measurement resolution set to 2.5 cm, cone number 3 and maximum depth of 40 cm.

After collecting the penetration resistance data in the field, information was transferred to the equipment’s accompanying software. The software allowed us to view, analyze and store data in the computer, as well as export it to other devices.

### Preparing maps for kriging

Field-collected data and their coordinates were exported to the ArcGIS software (ArcMap 10.2). Data interpolation was performed by simple kriging with the use of geostatistics techniques, including generating maps that represented resistance rates of evaluated areas.

Kringing is based on regression analysis techniques, according to which variance is estimated from a previous model, which takes into account the dependence of the available data in order to minimize error variance. Therefore, this method made it possible to estimate the conditions of locations within the geometry fields in the best possible way^31^.

The maps made by Kriging were configured in order to highlight tracks with penetration resistance greater than 4.0 MPa, which were considered to be compacted. This follows results observed by other authors^2,23,26,27^, who also considered values higher than the exposed as a limiting factor to root development of sugarcane.

### Preparation of operational/topographic maps

It was necessary to adapt the maps generated in ArcGIS to apply the results of subsoiling operations. For these technical adjustments, AutoCAD 2013 Software (Autodesk) was used. Zones for preparation were defined or adjusted considering the results of the interpolation and the topographic conditions of the terrain, such as levels and high traffic zones.

### Survey of costs

We made an effort to raise information about costs involved with the method used to diagnose the resistance of the soil against penetration. Costs included the price of acquisition of materials (packaging and supports) of the penetrometer equipment, the valuation of the labor employed to carry out the activity and the transportation costs for data collection.

In order to compare costs, we carried out quotations for the subsoiling operations, including labor, fuel and machinery depreciation. Those quotations were provided by three companies in the region who specialize in soil preparation services.

After collecting data related to all costs involved with the practice, we conducted a cost–benefit analysis in order to verify the economic viability of the analysis method. Moreover, the possibility of making economies in subsoiling operation in the evaluated areas was also determined, indicating the necessity for soil management.

## Results and discussion

The figures below show the values of soil resistance to mechanized penetration obtained in the field through the use of the penetrometer. These figures are based on a measurement resolution of 2.5 cm, covering depths from 0 to 40 cm, in the experimental areas under study.

**Figure 1:**
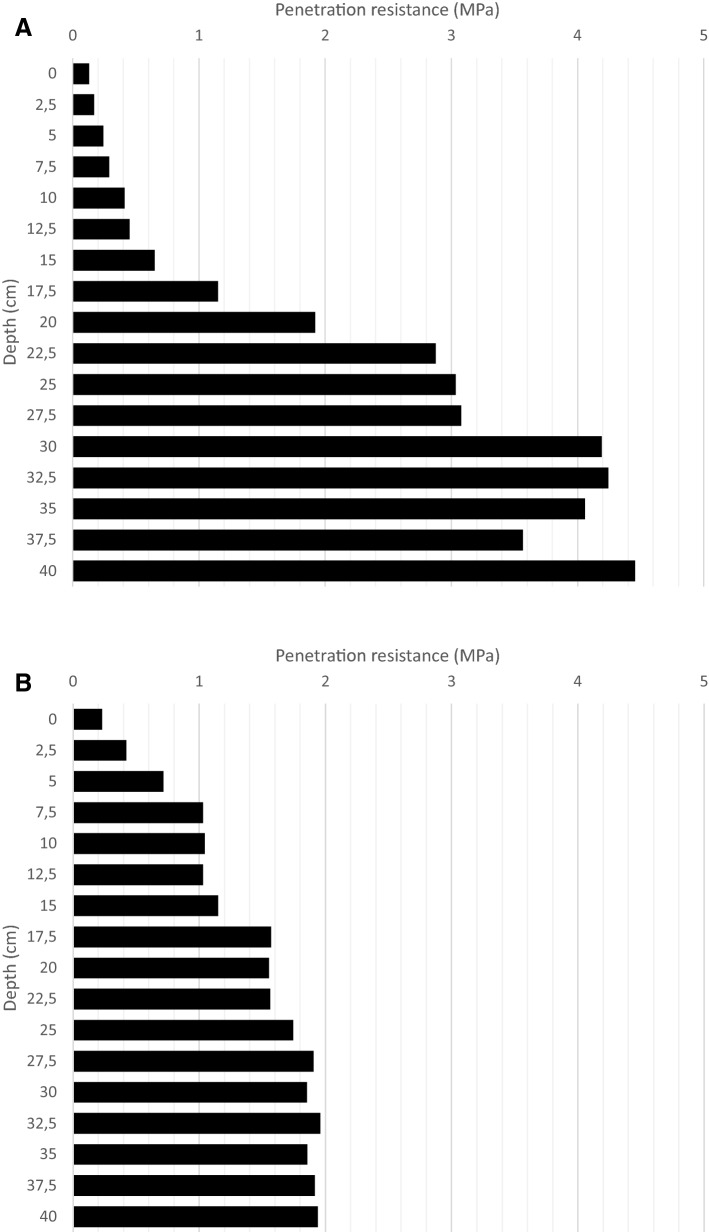
Resistance to root penetration (MPa) in soil cultivated with sugarcane in areas with compaction less than 4 MPa (**A**) and in areas with compaction greater than 4 MPa (**B**).

Based on the analysis presented in Fig. 1A, all sampled depths the values obtained were less than 4.0 MPa. Such a condition was obtained in most of the sampled areas. Figure 1B shows values above 4.0 MPa, which represents a small fraction of the total sampled area.

In Fig. 1A, B it is still possible to verify that penetration resistance values increase according to depth. This increase in resistance in relation to depth was also observed by other authors^2,26,32^.

A similar fact had already been reported by Carvalho et al.^33^ in a study that evaluated resistance against penetration in a system without soil overturning and with the presence of mulch. This condition can be attributed to more availability of organic matter in the superficial layer of the soil, and the mechanization process during fertilization and sowing^34^.

**Figure 2:**
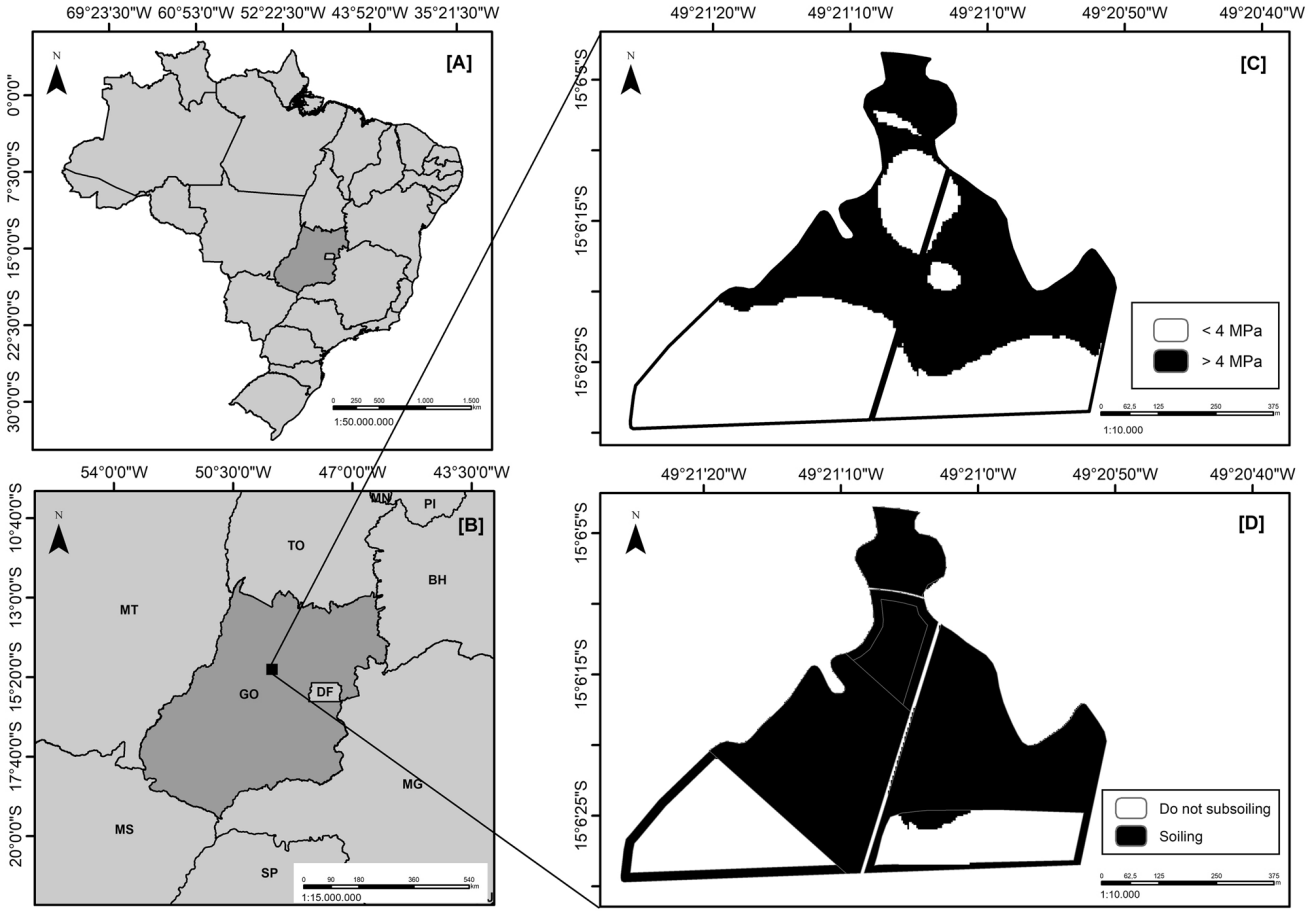
Kriging map of soil mechanical resistance to penetration in areas cultivated with sugarcane (**A**) and operational map for soil preparation with subsoiling (**B**). (Data and maps produced by the author. Generated by ArcGIS 10.2, http://www.esri.com/software/arcgis/arcgis-for-desktop).

Our study area is quite similar to other studies cited above. Mechanized harvesting keeps a large amount of vegetal cover on the ground. The cultivation and standard fertilization of sugar cane are carried out at a depth of up to 25 cm, which alters the superficial layer, presenting lower indices of root penetrations resistance. Figure 2 shows the kriging maps elaborated from data collected from soil resistance against penetration. These maps outline the conditions of the areas under study.

The existing terraced border areas were also used for deep preparation management, in addition to areas with penetration resistance greater than 4.0 MPa. Terrace areas have water retention and infiltration as one of their most important purposes. Therefore, it is extremely necessary that soils in these places have good permeability, as described by Machado and Wadt^35^. According to Rossini^32^, border areas are also classified as maneuver points, with greater probability of compaction.

Areas below and above 4.0 MPa were once again quantified through the use of topographic maps, as well as areas of borders and terraces. The maps enabled us to determine the final management framework, as it is shown in Table 1.

**Table 1:** Summary of assessed areas considering topographic adjustments.

FAZ	Nos.	Total area (ha)	Area relationship (ha)	
			< 4.0 MPa	> 4.0 MPa
A	1	8.56	8.56	0.00
	2	13.05	13.05	0.00
	3	12.52	12.52	0.00
	4	17.29	17.29	0.00
B	1	27.13	27.13	0.00
	2	23.89	23.89	0.00
	3	9.19	9.19	0.00
	4	25.03	25.03	0.00
	5	29.54	29.54	0.00
	6	21.88	21.88	0.00
	7	11.86	11.86	0.00
	8	2.03	2.03	0.00
C	1	48.06	48.06	0.00
	2	48.86	48.86	0.00
	3	47.97	30.62	17.35
	4	54.62	54.62	0.00
D	1	33.59	33.59	0.00
	2	40.76	22.19	18.57
	3	57.22	57.22	0.00
	4	24.30	24.30	0.00
	5	17.50	17.50	0.00
	6	72.93	72.93	0.00
	7	41.67	41.67	0.00
E	1	11.88	11.88	0.00
	2	28.59	28.59	0.00
	3	12.90	12.90	0.00
	4	49.39	49.39	0.00
	5	31.47	31.47	0.00
	6	35.94	35.94	0.00
	7	33.81	33.81	0.00
F	1	8.01	18.01	0.00
	2	7.95	67.95	0.00
	3	43.68	43.68	0.00
	4	15.66	15.66	0.00
Total (ha)		1038.73	1002.81	35.92
Total (%)		100%	96.54%	3.46%

By using the kriging methodology proposed in this work, it is plausible to obtain a reduction of 96.54% of the subsoiling operations in the studied area. The deep preparation activity being directed only to Fazenda C canopy 3 and Fazenda D 2, which presented higher values than the adopted critic (< 4.0 MPa). However, considering the operational practice of subsoiling activity in the field, topographic maps were utilized in order to indicate where to carry out subsoiling operations, as shown in Fig. 2B.

One of the most important purposes of terrace areas is the retention and infiltration of water. Thus, it is extremely necessary that the soils in these places have good permeability, as described by Machado and Wadt^35^. However, the border areas are classified as maneuver points where there is a greater likelihood of compaction^32^.

Areas below and above 4.0 MPa were once again quantified through the use of topographic maps, as well as areas of borders and terraces, which enabled us to determine the final management framework, as it is shown in Table 2.

**Table 2:** Summary of the areas evaluated considering topographical adjustments.

Farm	Number	Total area (ha)	Area relationship (ha)	
			Do not subsolate	Subsolate
A	1	8.56	5.79	2.77
	2	13.05	10.34	2.71
	3	12.52	9.46	3.06
	4	17.29	12.91	4.38
B	1	27.13	16.68	10.45
	2	23.89	15.73	8.16
	3	9.19	5.05	4.14
	4	25.03	16.07	8.96
	5	29.54	19.28	10.26
	6	21.88	14.58	7.30
	7	11.86	7.81	4.05
	8	2.03	1.40	0.63
C	1	48.06	36.51	11.55
	2	48.86	38.31	10.55
	3	47.97	24.36	23.61
	4	54.62	36.16	18.46
D	1	33.59	23.35	10.24
	2	40.76	19.01	21.75
	3	57.22	47.17	10.05
	4	24.30	19.88	4.42
	5	17.50	13.50	4.00
	6	72.93	63.28	9.65
	7	41.67	32.32	9.35
E	1	11.88	6.60	5.28
	2	28.59	22.46	6.13
	3	12.90	8.67	4.23
	4	49.39	41.45	7.94
	5	31.47	29.25	2.22
	6	35.94	30.99	4.95
	7	33.81	24.75	9.06
F	1	18.01	12.45	5.56
	2	67.95	57.01	10.94
	3	43.68	34.58	9.10
	4	15.66	12.22	3.44
Total (ha)		1038.73	769.38	269.35
Total (%)		100%	74.07%	25.93%

The need to perform the subsoiling operations was reduced by 74.07% through the use of the methodology proposed in this paper, even when considering terraces and borders as staging areas. According to quotes carried out in 2017 with three regional companies specialized in soil preparation services, the cost of operation per hectare—including labor, fuel and machinery depreciation—was US$ 63.75. From these data and financial quotes with real need for deep tillage (subsoiling), it was possible to achieve a savings of US$ 49,045.07. The quotation of the Brazilian currency (Brazilian real) of December 29, 2017 was used for the cost data of the operation.

Finally, it was possible to verify that net performance savings with management recommendation in the study area amounted to US$ 45,442.68. This figure is based on discounting the costs involved with the method used to diagnose the resistance of the soil to penetration, which amounted to US$ 3602.38. Our results show the feasibility and importance of the diagnosis in the area for carrying out preparation only where it is really necessary.

## Conclusions

1. The methodology for assessing resistance to soil penetration using a penetrometer locates compacted and non-compacted areas, enabling decisions about subsoiling operations aimed at suppressing deep preparation in unnecessary places.
2. In the areas studied, penetration resistance values increased in the function of depth that demonstrates, in general, subsurface layers have higher rates of resistance, even if they do not reach critical values.
3. By using kriging maps it was possible to verify that there has been a 96.54% reduction of the subsoil area, since only 35.92 ha from the total area of 1038.73 ha showed values higher than the critical standard adopted (< 4.0 MPa), requiring deep tillage operation.
4. Topographic maps allow for more precision in the applicability of operations in the field. This is the case when considering areas with values higher than the critical standard, or in the case of borders and terraces. Moreover, these maps also allow for a satisfactory reduction of 74.07% of the subsoiling practice when we consider that operations were deemed unnecessary in 769.38 ha out of the total sampled area.
